# Omission of Planning CT Reduces Patient Radiation Exposure during CT-Guided Bone Marrow Biopsy and Aspiration

**DOI:** 10.3390/tomography7040062

**Published:** 2021-11-08

**Authors:** Robert Devita, Kaushik Chagarlamudi, Jared Durieux, David Jordan, Brian Nguyen, Jon Davidson, Vijaya Kosaraju, Salim Abboud

**Affiliations:** 1 University Hospitals Cleveland Medical Center, Case Western Reserve University, Cleveland, OH 44106, USA; robert.devita@uhhospitals.org (R.D.); jared.durieux@uhhospitals.org (J.D.); david.jordan@uhhospitals.org (D.J.); jon.davidson@uhhospitals.org (J.D.); vijaya.kosaraju@uhhospitals.org (V.K.); salim.abboud@uhhospitals.org (S.A.); 2 School of Medicine, Case Western Reserve University, Cleveland, OH 44106, USA; bxn121@case.edu

**Keywords:** radiation exposure, quality improvement, CT-guided procedures

## Abstract

The purpose of this study is to evaluate the impact of eliminating a preprocedural planning computed tomography during CT-guided bone marrow biopsy on the technical aspects of the procedure, including patient dose, sample quality, procedure time, and CT fluoroscopy usage. Retrospective analysis of 109 patients between 1 June 2018 and 1 January 2021 was performed. Patients were grouped based on whether they received a planning CT scan. Relative radiation exposure was measured using dose-length product (DLP). Secondary metrics included number of CT fluoroscopic acquisitions until target localization, total number of CT fluoroscopic acquisitions, biopsy diagnostic yield, and procedure time. A total of 43 bone marrow biopsies with planning CT scans (Group 1) and 66 bone marrow biopsies without planning CT scans (Group 2) were performed. The average total DLP for Group 1 and Group 2 was 268.73 mGy*cm and 50.92 mGy*cm, respectively. The mean radiation dose reduction between the groups was 81% (*p* < 0.0001). Significantly more CT fluoroscopy acquisitions were needed for needle localization in Group 2 than Group 1 (*p* < 0.0001). Total number of CT fluoroscopy acquisitions was four for Group 1 and eight for Group 2 (*p* = 0.0002). There was no significant difference between the groups in procedure time or diagnostic yield. Patients without a planning CT scan received more fluoroscopic CT acquisitions but overall were exposed to significantly less radiation without an increase in procedure time.

## 1. Introduction

CT (computed tomography) has become an important image-guidance modality for many types of tissue sampling, including bone marrow biopsy and aspiration [1]. Several recent studies have shown that radiologists are performing more of these procedures for a variety of reasons, including patient obesity, prior failed bedside attempt, and sedation requirements, among other causes [1,2]. However, there is no standardized protocol for the use of CT while performing a bone marrow biopsy. Depending on user preference, a preprocedural planning scan (PPS) may be obtained for localization purposes, followed by limited CT fluoroscopy to guide needle placement. 

Radiologists should endeavor to reduce unnecessary radiation exposure while performing CT-guided procedures. Prior studies suggest that more than 90% of the patient’s absorbed dose during CT-guided procedures may be administered during the PPS [3]. If a PPS is deemed necessary by the radiologist, methods to limit radiation exposure include reducing the craniocaudal scan length (*z*-axis), increasing the pitch, decreasing the photon fluence (mA), and/or decreasing the beam energy (kVp) [2]. To our knowledge, the utility of a PPS for bone marrow biopsies has not been described. In our practice, some operators use a PPS, based on various training backgrounds. The use of a PPS for BMB has also been demonstrated at other institutions. For example, in a recent large-scale study comparing the biopsy yield of CT-guided vs. blind BMB, all CT-guided procedures utilized a PPS [4]. Therefore, the use of a PPS for CT-guided BMB is reported both in the published literature as well as commonly used in our institution’s experience. Its utility is not self-evident, however. The purpose of this study is to evaluate the impact of eliminating the PPS on various technical aspects of CT-guided BMB, including total radiation dose, changes in utilization of intermittent CT fluoroscopy, procedure time, and biopsy quality.

## 2. Materials and Methods

This retrospective HIPAA-compliant study was approved by the Institutional Review Board. Procedure codes from departmental Picture Archiving and Communication Systems (PACS) were searched to find all patients who underwent a CT-guided bone marrow biopsy performed at a single regional hospital at our institution by two attending radiologists from 1 June 2018 to 1 January 2021. Patients were included in the study if they underwent a CT-guided bone marrow biopsy in a prone position that included both dose reporting information and stored procedural images within our departmental PACS. A total of 120 CT-guided bone marrow biopsies were performed. Eleven patients were excluded from the study. Ten patients did not have images stored in PACS. One patient’s bone marrow biopsy was performed in the right lateral decubitus position. 

Our hospital began adoption of a CT-guided marrow biopsy protocol that excluded the PPS starting in Fall 2018. All patients prior to adoption of this protocol received a PPS at the start of the marrow biopsy procedure, followed by intermittent CT fluoroscopy for subsequent needle guidance. Bone marrow biopsies performed after the adoption of this protocol did not receive a PPS. Patients that received a PPS scan were assigned to Group 1 and patients who did not receive a PPS were assigned to Group 2. For patients in Group 2, the posterior superior iliac spine was palpated and marked by the radiologist before obtaining the first set of intermittent CT fluoroscopy images. The images obtained during each procedure were stored in our departmental PACS. For each case, dose length product (DLP), number of intermittent CT fluoroscopy acquisitions until localization of the targeted posterior ilium entry site, total number of intermittent CT fluoroscopy acquisitions, skin to bone distance, and procedure time were recorded. Relative radiation exposure to the patient was approximated using DLP. Localization of the target site was defined as the first image in which a needle was visualized within the subcutaneous soft tissues. Each intermittent CT fluoroscopy acquisition produced three contiguous axial CT images with 2.5 mm thickness at a fixed DLP of 6.37 mGy*cm. Starting procedural time for patients in Group 1 was defined as the time of the PPS. Starting time of the patients in Group 2 was defined as the time of the first CT fluoroscopy image. Ending procedural time for both Group 1 and Group 2 was defined by the time of the final CT fluoroscopy image. A retrospective chart analysis was performed regarding pertinent data, including body mass index and gender, as well as pathology results to ensure a proper sample was obtained. 

For the statistical analysis, continuous variables were described using the median and interquartile range. Discrete variables were described using frequency (*n*) and percentages (%). Differences between groups were computed using Mann–Whitney U-Test (WMW) and χ2 or Fisher’s exact tests for discrete variables. *p*-value less than alpha < 0.05 is considered statistically significant. All analyses were conducted using SAS 9.4 (SAS Inc., Cary, NC, USA).

## 3. Results

The median age of our sample was 69 (IQR: 57, 77) years old. Fifty-six percent (*n* = 61) were male and the median BMI was 28.5 kg/m^2^ (IQR: 23.1, 35.3). The median skin-to-bone measurement was 3.2 cm (IQR: 2, 5), the total median number of acquisitions was 7 (IQR: 4, 9), and the median procedure time was 13 (IQR: 9, 18). There were no significant differences in patient BMI, patient age, procedure time, and skin-to-bone distance (Table 1).

Median DLP in Group 1 was 268.73 (IQR: 143.05, 492.56) and in Group 2 was 50.92 (IQR: 31.83, 70.02) (*p* < 0.001). Median number of CT fluoroscopy acquisitions to localization in Group 1 was 1 (IQR: 0, 1) and in Group 2 was 3 (IQR: 2, 5) (*p* < 0.0001). Median total number of CT fluoroscopy acquisitions in Group 1 was 4 (IQR: 3, 8) and in Group 2 was 8 (IQR: 5, 11) (*p* = 0.0002). The results of the study are summarized in Table 1.

## 4. Discussion

A PPS scan is commonly obtained prior to CT-guided procedures for localization purposes [5,6,7]. It may provide valuable information for the radiologist, but can also contribute substantially to a patient’s radiation dose during CT-guided procedures, particularly when the remainder of the procedure is performed with intermittent CT fluoroscopy [3,6,7]. Multiple techniques have been previously described to lower the dose of PPS, including shortening the scan length and reducing the beam energy [3]. When an area of interest is anatomically consistent, ostensibly, the utility of PPS may decrease and the entirety of the procedure can be performed with intermittent CT fluoroscopy. The purpose of this study was to determine if eliminating the PPS reduced patient absorbed dose without compromising the technical aspects of the procedure. Our results demonstrate an 81% decrease in radiation dose with the elimination of the PPS, which is similar to previous studies showing approximately 90% of the absorbed patient dose occurs in the planning stage [3,7]. Furthermore, there were no complications in either group, and all obtained samples yielded diagnostic results, suggesting procedure efficacy and safety were not compromised by forgoing the PPS. 

Secondary endpoints of the study were the number of CT fluoroscopy acquisitions until target localization, procedure time, and total number of CT fluoroscopy acquisitions. Procedure time did not vary significantly between the groups. Our results show significantly more CT fluoroscopy acquisitions were used to localize the target site as well as for the entire procedure for patients that did not receive a PPS. Since each CT fluoroscopy acquisition on our CT unit exposed the patient to a relatively small dose of 6.37 mGy*cm, total radiation dose to the patient was still markedly lower in Group 2 despite additional fluoroscopy acquisitions. Since identifying the optimal access site is largely performed on physical examination, Group 2 required about three CT fluoroscopy acquisitions on average compared to one for Group 1, i.e., for Group 2, the information Group 1 received via the PPS was effectively replaced via two additional fluoroscopy acquisitions. 

Our CT fluoroscopy acquisitions are obtained via a foot pedal within the procedure room behind a moveable, radiation-shielded wall. While we did not directly measure radiation exposure of the radiologist, the increased number of intermittent CT fluoroscopy acquisitions could potentially increase scatter dose to the radiologist. Indeed, operator dose is an important recognized limitation of intermittent CT fluoroscopy since the operator is usually required to be in the procedure suite at the time of image acquisitions [6,7]. Our results suggest that CT-guided bone marrow biopsies that rely entirely on intermittent CT fluoroscopy by omitting the PPS may increase operator radiation dose by increasing the utilization of intermittent CT fluoroscopy. Interventionalists considering forgoing the PPS should therefore consider steps to reduce their radiation exposure, such as adding additional shielding in the intervention suite or increasing operator distance from the scanner when obtaining fluoroscopic acquisitions. 

Our study has several limitations. First, all of the patients in the study were in the prone position, and therefore these findings may not apply to CT-guided marrow biopsies performed with different or atypical patient positioning. Second, we did not use a CT topogram to aid identification of the entry site for Group 2. A planning topogram could potentially minimize the amount of CT fluoroscopy acquisitions needed to identify an ideal target site even when not performing a PPS.

Finally, in this study, DLP was used as a surrogate for radiation dose, though there are several limitations. DLP is a measurement of radiation output from the scanner (and energy imparted to the patient). It varies considerably based on the selected scan length, which is often a subjective assessment by the technologist. DLP does not account for patient size, although in our sample, there were no significant differences in BMI between the groups, suggesting that imparted energy for the two groups would show the same trend as absorbed dose and effective dose. The same anatomical region was scanned in all subjects, so the same radiosensitive organs and tissues were exposed. Although DLP does not provide accurate absolute radiation dosimetry, it was used as a readily available, sufficient surrogate to compare the subject groups since the relative difference in exposure was the parameter of interest.

## 5. Conclusions

In conclusion, elimination of a PPS prior to bone marrow biopsy significantly reduced the patient’s radiation dose without any significant negative impact on the technical aspects of the procedure or decreasing sample quality. Employing this change in routine practice is compatible with the patient radiation protection principles of justification and limitation of doses, and thus, in the appropriate patient population, the radiologist should consider performing bone marrow biopsies without a PPS.

## Figures and Tables

**Table 1:** Characteristics of Bone Marrow Biopsy Patients.

	All Participants (*n* = 109)	Preprocedural Planning Scan (*n* = 43)	No Pre-Procedural Planning Scan (*n* = 66)	
**Characteristics**	Median (IQR)/Frequency (%)	Median (IQR)/Frequency (%)	Median (IQR)/Frequency (%)	*p*-value
Age (years)	69 (57, 77)	70 (53, 80)	69 (59, 76)	0.25
Female Gender	48 (44.04)	21 (48.84)	27 (40.91)	0.42
BMI (kg/m^2^)	28.5 (23.1, 35.3)	28.5 (22.5, 39)	28.55 (24.4, 35.2)	0.46
Skin to Bone (cm)	3.2 (2, 5)	3.3 (2, 5.4)	3.05 (2, 4.4)	0.29
DLP Total	76.38 (44.56, 170.03)	268.73 (143.05, 492.56)	50.92 (31.83, 70.02)	<0.0001
Total number of CT Fluoroscopy Acquisitions	7 (4,9)	4 (3, 8)	8 (5, 11)	0.0002
Number of CT Fluoroscopy Acquisitions to Localization	2 (1, 4)	1 (0, 1)	3 (2, 5)	<0.0001
Procedure Time	13 (9, 18)	13 (10, 19)	12.5 (8, 18)	0.09

## Data Availability

Institutional RedCap.
